# When Our “Best” Isn’t Good Enough: Asleep Paralyzed Fiberoptic Intubation After a Failed Awake Fiberoptic at an Outside Hospital

**DOI:** 10.7759/cureus.95439

**Published:** 2025-10-26

**Authors:** James Chen, Benjamin D Brakke, Timon J Higgins, Bradly J Narr

**Affiliations:** 1 Anesthesiology and Perioperative Medicine, Mayo Clinic, Rochester, USA

**Keywords:** airway anatomy, difficult airway management, failed intubation, fiberoptic intubation, video laryngoscopy

## Abstract

A 72-year-old, 127-cm, 53-kg female presented to our hospital after her surgery was canceled at an outside hospital due to multiple failed awake fiberoptic intubation attempts. She required a total abdominal hysterectomy, bilateral salpingectomy, and pelvic lymph node dissection for endometrial cancer. Given the prior failed awake attempts, we performed an asleep, paralyzed, combined video laryngoscope and fiberoptic oral intubation. The decision-making, actions, and risk mitigation provide an interesting case for discussion.

## Introduction

Truly difficult airways have become rare with the ubiquity of advanced airway techniques and equipment, specifically video laryngoscopes and fiberoptic scopes. The modern incidence of “cannot intubate, cannot ventilate” situations is estimated to be 0.0019% to 0.04% of all airways [1]. Unfortunately, even airways that appear straightforward can end in loss of the airway, hypoxia, cardiac arrest, or anoxic brain injury.

Developed in the 1990s and most recently updated in 2022, the American Society of Anesthesiologists (ASA) Difficult Airway Algorithm [2] provides guidance to improve patient safety when anesthesiologists anticipate or encounter difficulty with intubation, supraglottic airway placement, or ventilation. If difficulty is anticipated, the algorithm encourages maintaining spontaneous ventilation until the airway can be safely secured. In addition, it advocates limiting attempts and aborting if unsuccessful, because repeated attempts may further compromise the airway through complications such as swelling or bleeding.

This case report was previously presented at the Midwest Anesthesia Residents Conference (MARC) on April 6, 2024, as well as at the ASA meeting on October 19, 2024.

## Case presentation

A 72-year-old, 127-cm, 53-kg woman presented on the day of surgery for a robotic total abdominal hysterectomy, bilateral salpingectomy, and pelvic lymph node dissection for endometrial cancer. At another institution, the patient could not be intubated after three awake fiberoptic attempts despite a recognized difficult airway. The patient was referred to our hospital for further management of her difficult airway.

Her airway issues resulted from severe juvenile rheumatoid arthritis, two cervical spine fusions with severely limited head and neck motion restricting oropharyngeal and laryngeal axial alignment, temporomandibular joint immobility with a small mouth opening, a large tongue, and short stature. The outside hospital airway note mentioned that a gastroenterology upper endoscopic procedure had to be aborted due to the scope “hitting something.” Our airway exam was not reassuring: interincisor distance less than 2.5 cm, little to no neck extension, upper lip bite test grade 3, and thyromental and hypothyroid distance less than three fingerbreadths. No recent lateral neck imaging was available.

The outside hospital awake fiberoptic attempts were induced with topical lidocaine, nasal oxymetazoline, intravenous glycopyrrolate 0.2 mg, midazolam 1 mg, and propofol 20 mg. The first attempt obtained a Cormack-Lehane grade 4 view using a fiberoptic scope placed orally. Due to gagging, coughing, hypoxia, and difficulty ventilating, this attempt was aborted. The second attempt entered the left nare, where the epiglottis but no vocal cords were seen (grade 3b). Attempts to drive the scope under the epiglottis were unsuccessful and resulted only in esophageal visualization. They aborted during the third awake fiberoptic attempt in the right nare due to mounting secretions and tissue edema, noting airway collapse along with loss of epiglottic visualization during each respiratory cycle. Failure to secure the airway resulted in case cancellation.

**Figure 1:**
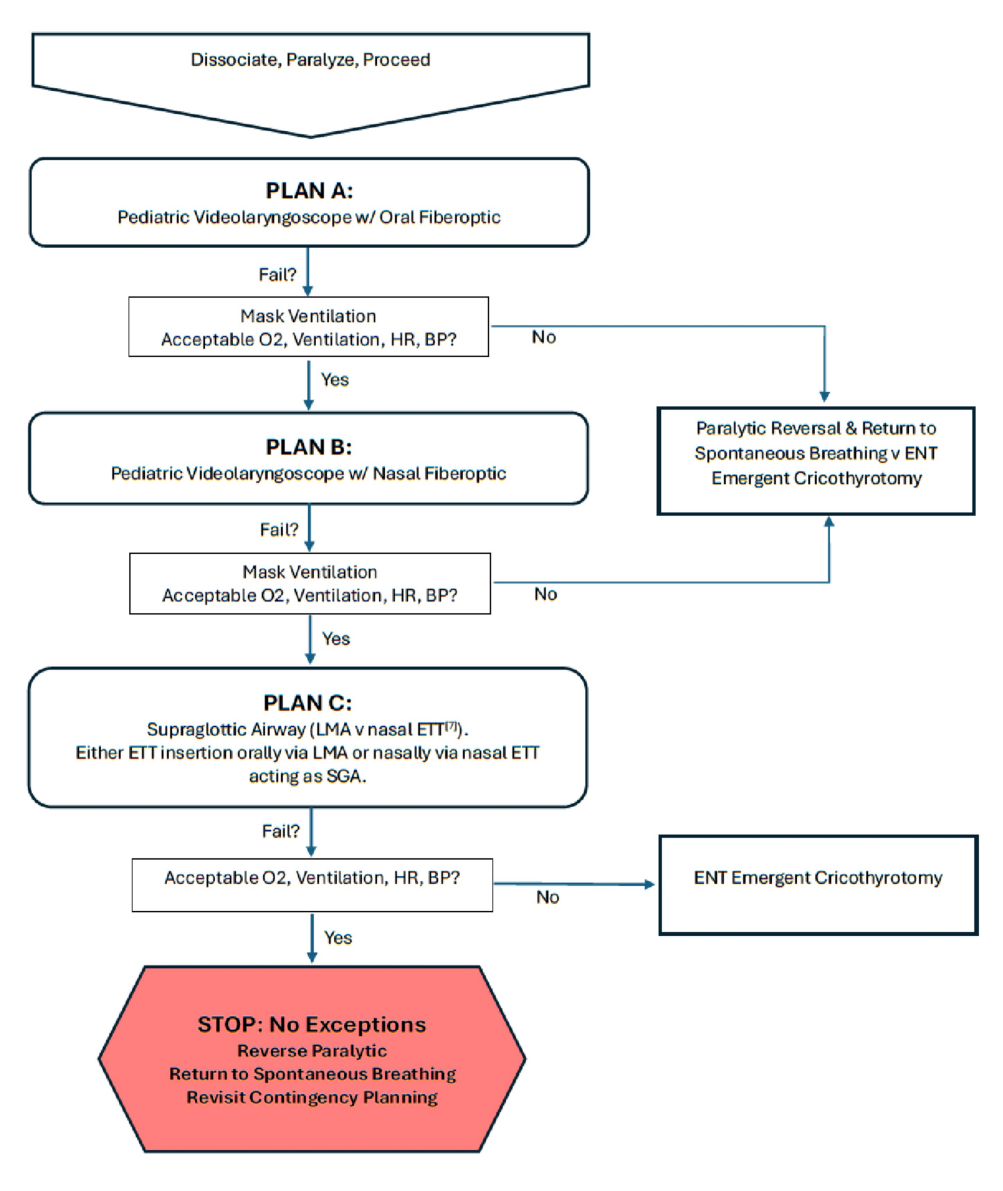
Our Airway Plan and Algorithm ETT: endotracheal tube, SGA: supraglottic airway, LMA: laryngeal mask airway, ENT: otolaryngology, O2: oxygen saturation, HR: heart rate, BP: blood pressure.

We focused on an asleep, paralyzed technique with three planned approaches. Plan A would use a pediatric video laryngoscope with oral fiberoptic endotracheal tube placement. Plan B would be nasal fiberoptic endotracheal tube placement assisted by video laryngoscopy. Plan C would involve placing an intubating supraglottic airway with subsequent fiberoptic endotracheal tube placement. Should her small mouth opening limit supraglottic airway placement, we could use a nasally placed endotracheal tube or trumpet to ventilate while awaiting the return of spontaneous ventilation (Figure 1). These attempts were performed in the supine position, with the operating room table positioned as low as possible to allow for better fiberoptic procedural ergonomics.

To help mitigate risk, we selected amnestic induction agents that allowed spontaneous ventilation and a reversible paralytic agent; should we fail to establish an airway, we could reverse the paralytic and promote more rapid spontaneous ventilation. We appointed a senior anesthesia resident, fully briefed on the airway plan, who would remind us to abort if additional airway attempts were made beyond “Plan C.” An otolaryngology (ENT) physician was present in case an emergent cricothyrotomy was needed. After exhaustive discussions with the patient and surgical staff concerning the risks and management, we proceeded to the operating room.

We paused operating room activities for an anesthesia timeout, detailed the anesthesia plan, and answered any questions. The ENT physician examined the front of the neck and confirmed the feasibility of an emergent surgical airway should it be required. We proceeded with induction: midazolam 2 mg, ketamine 40 mg, dexmedetomidine 32 mcg, and propofol 20 mg followed by rocuronium 50 mg (0.93 mg/kg). One-handed mask ventilation was difficult and required progressive escalation to oral airway, two-handed jaw thrust, and nasal trumpet. Airway resistance was high, but adequate tidal volumes (250-300 cc/breath) were achieved.

Our first attempt using a pediatric-sized video laryngoscope yielded a grade 3b view. The epiglottis rested fixed on the posterior arytenoids with no view of the glottic opening. Driving the fiberoptic scope beneath the epiglottis initially resulted in esophageal visualization. Upon further inspection directly under the epiglottis, we encountered a blind tunnel of tissue that, after several adjustments, led to the glottic opening and into the trachea. A 6.0 mm lubricated endotracheal tube was threaded off the fiberoptic scope and into the trachea without difficulty. Total airway manipulation time approximated one minute, and the patient did not experience oxygen desaturation.

After roughly three hours, the surgical team completed the surgery without incident. Planning for extubation followed a normal cuff leak test, train-of-four monitoring, and reversal of rocuronium-induced paralysis with sugammadex dosed at 4 mg/kg. The patient returned to spontaneous breathing with appropriate tidal volumes, and after she followed commands, the anesthesia team extubated her without any complications. The following day, the patient did not recall any memory of the intubation.

## Discussion

This case demonstrates the complexities and risks of managing a truly difficult airway, even in a controlled setting with extensive preparation. While we achieved a successful outcome, key elements of the case highlight critical learning lessons.

Our decision to proceed with an asleep and paralyzed intubation diverged from the ASA Difficult Airway Algorithm [2], which advocates maintaining spontaneous ventilation during difficult intubations. After multiple failed awake fiberoptic attempts at the outside hospital, and only after careful deliberation of the risks and benefits of various approaches, we believed that the asleep, paralyzed approach would be more effective. Interestingly, a retrospective Norwegian study of 833 awake fiberoptic intubations between 2011 and 2021 reported 29 failed attempts; 21 of these were secured with direct or video laryngoscopy after initiating general anesthesia, nine of which received a muscle paralytic [3].

Though several experienced anesthesiologists questioned the outside hospital’s airway expertise, an awake fiberoptic approach, even with the depth and breadth of in-house airway expertise-though consistent with the ASA Difficult Airway Algorithm-could not guarantee a materially superior result, primarily due to the anatomic limitations (severely limited neck flexion and head extension due to two cervical spine fusions, small mouth opening, large tongue, etc.), in addition to dynamic airway collapse with loss of epiglottic visualization and increased secretions on successive airway attempts per the outside hospital airway note. In other words, we needed to optimize our first attempt, potentially with more manipulation than could be tolerated during an awake attempt.

Our approach posed inherent risks. Despite several studies describing the feasibility of emergent reversal of neuromuscular blockade [4,5], there was no guarantee of return to spontaneous ventilation with our combination of multiple induction agents. Furthermore, while a supraglottic airway served as a backup plan, the 2019 Closed Claims Analysis indicates that such devices frequently prove insufficient in “cannot intubate, cannot ventilate” situations [6]. Moreover, there was no guarantee that we would be able to place one through her small mouth opening; however, the backup would be to ventilate nasally via endotracheal tube [7] until the return of spontaneous ventilation. These considerations underscored the importance of meticulous preparation, including clear role assignments, detailed planning, limiting airway attempts, and insisting on otolaryngology presence for emergent intervention.

Despite these measures, we identified areas for improvement. For instance, we initially neglected to administer oxymetazoline and glycopyrrolate, which could have reduced secretions and enhanced visibility; both were eventually administered. This oversight, while not detrimental in this case, emphasizes the importance of checklists and structured anesthesia timeouts. From a systems perspective, better coordination could have improved the patient’s care. An otolaryngology evaluation days before surgery might have clarified the anatomy and allowed for contingency planning, such as consideration of an awake tracheostomy or extracorporeal membrane oxygenation (ECMO) pre-cannulation.

## Conclusions

Ultimately, our decision to use an asleep and paralyzed technique proved successful but not without controversy. One co-author of the Difficult Airway Algorithm summarized the outcome as “successful because it worked,” while another co-author noted, “You never want to burn a bridge,” emphasizing the importance of spontaneous ventilation. Although our approach carried inherent risks, we believe it was carefully calibrated against the likelihood of another failed awake fiberoptic attempt, along with another case cancellation in a patient with cancer. We should emphasize that divergence from the Difficult Airway Algorithm should not be the norm nor taken lightly.

This case highlights the importance of flexible decision-making, individualized precision medicine, robust preparation, risk mitigation, and continuous learning in airway management. By critically evaluating our approach and outcomes, we aim to improve systems and protocols to better serve future patients.
